# Preparation and characterization of layered double hydroxide (LDH) films with varying divalent cation species on Al–Si–Cu alloys by steam coating

**DOI:** 10.1039/d5ra09199c

**Published:** 2026-04-13

**Authors:** Io Matsui, Yuki Atsuumi, Hikari Ouchi, Kota Fukuhara, Takahiro Ishizaki

**Affiliations:** a Materials Science and Engineering, Graduate School of Engineering and Science, Shibaura Institute of Technology Tokyo 135-8548 Japan; b College of Engineering, Shibaura Institute of Technology Tokyo 135-8548 Japan ishizaki@shibaura-it.ac.jp

## Abstract

Aluminum alloys are widely utilized in various industries due to their excellent mechanical and physical properties. However, the presence of alloying elements like Si and Cu in Al–Si–Cu alloys necessitates surface treatment to mitigate their inherent susceptibility to corrosion. In this study, layered double hydroxide (LDH) films containing different divalent cations—namely, Mg–Al, Co–Al, Ni–Al, and Zn–Al LDH films—were prepared on Al–Si–Cu alloy substrates using the steam coating method to investigate the influence of the cation species on corrosion protection performance. The films were characterized by X-ray diffraction (XRD), scanning electron microscopy (SEM), energy-dispersive X-ray spectroscopy (EDS), and Fourier-transform infrared spectroscopy (FT-IR). Structural analyses indicated the formation of predominantly nitrate-based LDH films with partial carbonate incorporation, while the Zn–Al system formed a composite film of LDH and ZnO. SEM observation revealed that, despite having the greatest thickness, the Mg–Al film exhibited significant surface cracks due to its rapid growth rate. Corrosion resistance was quantitatively evaluated through potentiodynamic polarization curves, electrochemical impedance spectroscopy (EIS) measurements, and long-term immersion tests in NaCl solution. Based on the mean corrosion current density (*i*_corr_) and ion-release behavior, the overall order of corrosion resistance was determined to be Ni–Al > Co–Al > Mg–Al > Zn–Al LDH. Notably, the superior electrochemical barrier performance of the Ni–Al film was evidenced by its low *i*_corr_ and high resistance values, which remained consistent across replicate tests. In contrast, the Co–Al LDH film exhibited *i*_corr_ values comparable to those of the bare alloy, but with a large standard deviation, indicating inconsistent protective performance despite its dense microstructure. Conversely, the high variability and lower protection of the Mg–Al film were attributed to the presence of large cracks, which acted as electrolyte pathways. These findings established that the compactness and structural integrity of the LDH film, both of which are primarily dictated by the divalent cation species, are the decisive factors governing the corrosion protection of Al–Si–Cu alloys.

## Abstract 2

Aluminum alloys are widely utilized in various industries due to their excellent mechanical and physical properties.

## Graphical abstract

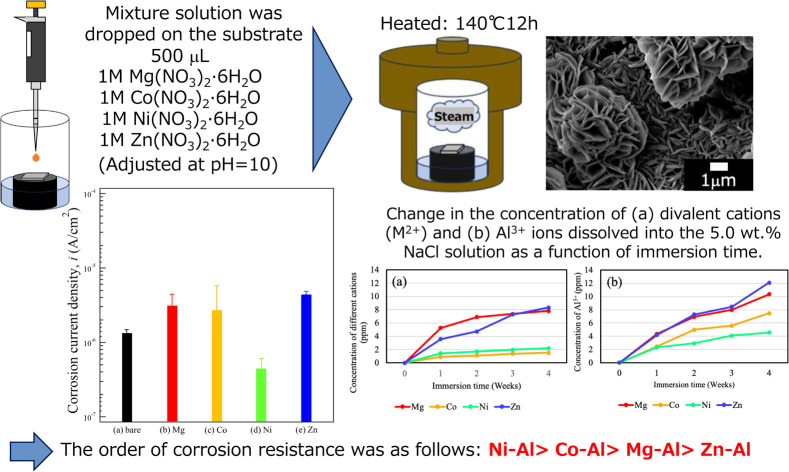

## 1. Introduction

Aluminium alloys possess excellent properties such as low density, high strength, and high electrical conductivity, which allow them to be widely used as structural materials in transportation equipment, electrical devices, marine structures, and aerospace applications.^1–5^ In particular, the demand for aluminium alloys has been increasing due to the ongoing emphasis on weight reduction to mitigate CO_2_ emissions. However, aluminium alloys exhibit a relatively low corrosion potential and high surface energy, making them susceptible to electrochemical corrosion in humid environments. This susceptibility is further exacerbated in chloride-containing environments, where localized corrosion such as pitting and intergranular corrosion is likely to occur.^6–10^ Al–Si–Cu alloys (ADC12), which are commonly used as aluminium die-casting materials, are known to exhibit particularly low corrosion resistance due to the presence of alloying elements such as Si and Cu.^11^

To suppress the progression of corrosion, various surface treatments, including anodizing, chemical conversion, and electroplating, have been employed. However, these conventional treatments require stringent bath management and waste-solution processing, which present significant environmental burdens.^12–15^ To address these issues, a low-environmental-impact surface treatment technique, known as the steam coating method, has been developed.^16^ The steam coating method is a surface treatment technique in which metals are reacted with steam inside a heated, sealed vessel to form oxide or hydroxide films on the metal surface.^16^

Layered Double Hydroxide (LDH) is one of the coatings that can be produced using this technique. The general formula of LDH is expressed as [M_1−*x*_^2+^M_*x*_^3+^(OH)_2_]^*x*+^[A_*x*/*n*_^*n*−^]^*x*−^·*m*H_2_O (0.17 ≤ *x* ≤ 0.33), and the structure is analogous to that of brucite, Mg(OH)_2_. Here, M^2+^, M^3+^, and A^*n*−^ represent divalent metal cations (*e.g.*, Mg^2+^, Zn^2+^, Cu^2+^, Mn^2+^), trivalent metal cations (*e.g.*, Al^3+^, Co^3+^, Fe^3+^), and *n*-valent anions (*e.g.*, NO_3_^−^, Cl^−^, CO_3_^2−^, PO_4_^3−^), respectively.^17–19^ Because a portion of the divalent metal ions in the hydroxide layers is substituted by trivalent cations, the host layers carry a positive charge.^20,21^ To compensate for this charge, interlayers consisting of anions and interlayer water molecules are alternately stacked between the positively charged layers. The anions present in the interlayer region can be exchanged with other guest anions, a phenomenon known as intercalation. Intercalation is known to occur more readily for anions with higher charge density and greater valence, meaning that such anions are more easily exchanged and captured within the interlayer galleries of LDH. Exploiting this functionality, corrosive species such as chloride ions (Cl^−^) can be selectively incorporated into the interlayer region, thereby suppressing the initiation of pitting corrosion and subsequent corrosion propagation, ultimately enhancing the corrosion resistance of the material.^22–24^ However, a systematic understanding of how the inherent thermodynamic properties of the divalent cation (M^2+^), such as their propensity for hydroxide formation, dictate the final microstructure and properties of steam-coated LDH films remains limited.

To address this critical knowledge gap, in this study, we employed the steam coating method to fabricate M^2+^–Al LDH films (M^2+^ = Mg, Co, Ni, Zn) on ADC12 alloy and evaluated their structural properties and their correlation with the standard Gibbs free energy of formation (Δ*G*^0^_f_) of the M^2+^ hydroxides. The resulting films were characterized in terms of their structure and composition using X-ray diffraction (XRD), field-emission scanning electron microscopy (FE-SEM), FE-SEM coupled with energy-dispersive X-ray spectroscopy (EDS), and Fourier-transform infrared spectroscopy (FT-IR). Furthermore, the corrosion resistance of the untreated substrate and various LDH coatings was systematically evaluated using potentiodynamic polarization tests, electrochemical impedance spectroscopy (EIS), and salt immersion tests conducted in a 5.0 wt% NaCl aqueous solution.

## 2. Experimental

Al–Si–Cu alloy specimens with dimensions of 20 × 20 × 2 mm (hereafter referred to as ADC12) were used in this study. The chemical composition of the alloy is listed in Table 1. As a pretreatment, the specimen surface was ground with waterproof abrasive papers (#400, #1200, and #2000) to remove the native oxide film and surface scratches, followed by ultrasonic cleaning in ethanol (99.5% purity) for 10 min at 42 kHz, and subsequently dried with nitrogen gas (99.5% purity).

**Table 1:** Chemical composition of ADC12 substrate. (wt%)

Si	Cu	Mg	Zn	Fe	Mn	Al
11.38	1.58	0.26	0.72	0.87	0.16	Bal.

The LDH films were grown *via* steam coating in a Teflon-lined stainless-steel autoclave (total volume: 100 mL). For the vapor source, 20 mL of ultrapure water (resistivity: 18.2 MΩ cm) was placed at the bottom, achieving a filling ratio of 20 vol%. To ensure that the film growth occurred exclusively in the gas phase, the specimen was mounted on a stainless-steel holder (wrapped with Teflon tape) and positioned horizontally, parallel to the liquid surface at a distance of approximately 1 cm above the water level; this setup ensured the specimen was exposed only to steam and not immersed in the liquid phase. The thermal profile was strictly controlled: the sealed autoclave was heated in an electric furnace to 140 °C at a constant ramp rate of 10 °C min^−1^, followed by a hold time of 12 h. After the reaction, the system was naturally cooled to room temperature. No mechanical stirring or agitation was applied during the entire steam coating process to maintain a static growth environment. Before the thermal treatment, 500 µL of the precursor solution (1.0 M nitrate salts (Mg(NO_3_)_2_), (Ni(NO_3_)_2_, Co(NO_3_)_2_, Zn(NO_3_)_2_), pH adjusted to 10 *via* dropwise addition of 1.0 M NaOH) was deposited onto the substrate surface. The pH of each precursor solution was precisely adjusted to 10 by the dropwise addition of 1.0 M NaOH as the base, using a micropipette under continuous stirring at 800 rpm. This adjustment was performed at room temperature (approx. 25 °C). After reaching the target pH, the slurry was aged for 30 min under stirring to ensure chemical homogeneity before being transferred to the autoclave. To minimize CO_2_/carbonate contamination, all precursor solutions were prepared using freshly boiled ultrapure water (to expel dissolved CO_2_), and the pH adjustment was carried out in a partially covered vessel to limit atmospheric exposure. These precautions ensure the formation of the desired LDH structure with minimal carbonate interference. Hereafter, the LDH samples prepared using Mg, Co, Ni, and Zn nitrate sources are referred to as Mg–Al, Co–Al, Ni–Al, and Zn–Al, respectively.

The crystalline phases of the fabricated films were identified using X-ray diffraction (XRD, Ultima IV, Rigaku Co., Cu Kα radiation, 40 kV, 40 mA). XRD patterns were obtained in the 2*θ* configuration. The surface and cross-sectional morphologies, and elemental distributions, were examined using field-emission scanning electron microscopy coupled with energy-dispersive X-ray spectroscopy (FE-SEM/EDS, JSM-IT300HR, JEOL Ltd, 30 kV). To prepare cross-sectional specimens, the samples were first embedded in resin and immobilized *via* vacuum drying. The cross-sections were then fabricated using an argon ion milling system (Cross-section Polisher, CP: IB-09010CP, JEOL Ltd) at an accelerating voltage of 6 kV for 7 h, with the ion beam irradiated from the lateral side of the resin-encapsulated sample. The chemical bonding states of the films were analysed by Fourier transform infrared spectroscopy (FT-IR, IRTracer-100, Shimadzu Corp.) using the attenuated total reflectance (ATR) method.

The corrosion resistance of the films was evaluated by potentiodynamic polarization and electrochemical impedance spectroscopy (EIS). For electrochemical measurements, the coated and uncoated Al alloy specimens were used as working electrodes, a Pt mesh as the counter electrode, and a saturated Ag/AgCl electrode as the reference electrode. The exposed area of the working electrode was strictly defined as 1.0 cm^2^ using a commercial electrolyte cell (EC-FLAT-TOY: TOYO Corporation, Tokyo Japan). Before the polarization measurements, the specimens were immersed in 5.0 wt% NaCl solution deoxygenated by nitrogen bubbling for 20 min, followed by open-circuit potential stabilization for 30 min. Potentiodynamic polarization curves were obtained by scanning the potential from −200 mV to +500 mV relative to the open-circuit potential at a scan rate of 0.5 mV s^−1^. During measurements, nitrogen gas was continuously flowed over the electrolyte surface to minimize oxygen ingress. All polarization measurements were conducted in 5 wt% NaCl aqueous solution at room temperature three times. Tafel fitting was performed within the range of ±50 to ±150 mV from the *E*_corr_ to ensure a linear Tafel region.

EIS measurements were conducted at open-circuit potential using the same electrode configuration. An AC amplitude of 10 mV was applied, and the frequency range was set from 10 mHz to 1 MHz. The acquired impedance spectra were analysed using equivalent circuit models implemented in ZView software (Scribner Associates). The EIS data were fitted using an equivalent circuit model. The goodness-of-fit (*χ*^2^) values were in the range of 10^−4^ to 10^−3^. The CPE exponents (^*n*^) were found to be 0.70 to 0.92, indicating the surface inhomogeneity of the electrodes. Salt immersion tests were performed using specimens with an exposed surface area of 1.0 cm^2^. The specimens were immersed in 50 mL of 5.0 wt% NaCl solution maintained at 35 °C for a total of 4 weeks. To quantitatively evaluate the concentration of metal ions released into the solution during immersion, inductively coupled plasma optical emission spectroscopy (ICP-OES, Agilent Technologies) was employed. Further details regarding the measurement and analysis of ICP are provided in the Experimental section of the SI (SI, S1).

## 3. Results and discussion

**Fig. 1:**
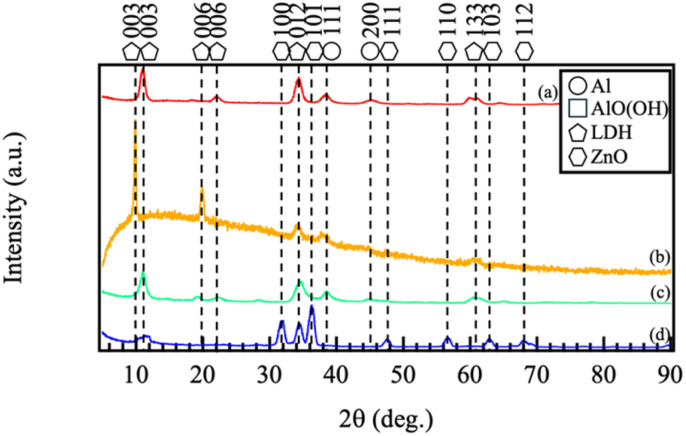
XRD patterns of the films prepared by steam coating at 140 °C for 12 h using an aqueous solution (pH = 10) containing 1.0 M of (a) Mg(NO_3_)_2_·6H_2_O, (b) Co(NO_3_)_2_·6H_2_O, (c) Ni(NO_3_)_2_·6H_2_O, or (d) Zn(NO_3_)_2_·6H_2_O.

Fig. 1 shows the XRD patterns of samples prepared by dropping 500 µL of 1.0 M aqueous solutions of (a) Mg–Al, (b) Co–Al, (c) Ni–Al, and (d) Zn–Al films. In the XRD patterns of all samples, peaks were observed at 2*θ* values ranging from approximately 10 to 11.5° (corresponding to the 003 reflection), as well as near 22°, 34°, and 61°, which correspond to the 006, 012, and 110 planes of the LDH structure. The *d*_003_ basal spacings calculated from these peak positions were approximately 0.76–0.85 nm. These values are intermediate between those reported for pure nitrate-type LDH (*ca.* 0.89 nm) and carbonate-type LDH (*ca.* 0.76 nm). Considering that the synthesis was conducted under atmospheric conditions, these results suggest that the films consist of LDH with mixed interlayer anions, where nitrate ions from the precursor are partially replaced by carbonate ions absorbed from the air.

For the sample prepared with Zn(NO_3_)_2_·6H_2_O (Fig. 1d), the 003 reflection of LDH was observed near 2*θ* = 10°, although it was broad. Additional peaks at approximately 2*θ* = 31.8°, 34.4°, 36.3°, 47.5°, 56.6°, 62.9°, and 67.9° were also detected, which are assigned to ZnO (JCPDS No. 36-1451). The formation of ZnO is likely due to the partial dehydration of Zn(OH)_2_ present during the reaction under the hydrothermal conditions. These results indicate that while Mg(NO_3_)_2_·6H_2_O, Co(NO_3_)_2_·6H_2_O, or Ni(NO_3_)_2_·6H_2_O yielded films predominantly composed of the LDH phase, the use of Zn(NO_3_)_2_·6H_2_O resulted in a mixed-phase film consisting of LDH and ZnO. This clearly demonstrates that the type of divalent cation (M^2+^) strongly influences the LDH formation kinetics and the final crystalline phases of the coating.

**Fig. 2:**
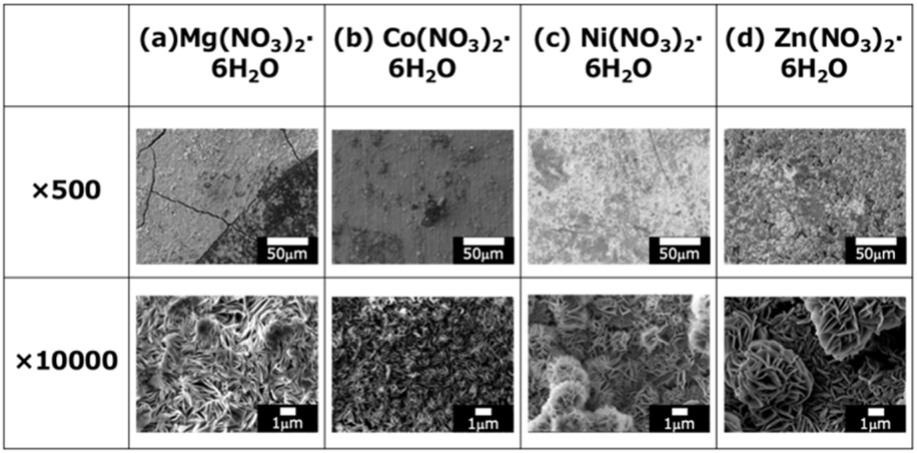
SEM images of the films prepared by steam coating at 140 °C for 12 h using an aqueous solution (pH = 10) containing 1.0 M of (a) Mg(NO_3_)_2_·6H_2_O, (b) Co(NO_3_)_2_·6H_2_O, (c) Ni(NO_3_)_2_·6H_2_O, or (d) Zn(NO_3_)_2_·6H_2_O.

Fig. 2 presents representative FE-SEM images of (a) Mg–Al, (b) Co–Al, (c) Ni–Al, and (d) Zn–Al films. Under all conditions, the formation of the characteristic LDH plate-like structure was confirmed.^25^ Furthermore, to ensure the morphological homogeneity across the substrate, supplementary FE-SEM images taken from different locations are provided in Electronic Supplementary Information Fig. S1. These supplementary figures confirm that the observed morphologies are representative and do not exhibit significant spatial dependence across the coating surface. Detailed examination of the FE-SEM images reveals distinct morphological features. In the Mg–Al (Fig. 2a) and Co–Al (Fig. 2b) samples, densely stacked plate-like crystals were predominantly observed. In contrast, the Ni–Al (Fig. 2c) and Zn–Al (Fig. 2d) samples exhibited plate-like structures assembled into a more defined flower-like morphology. This variation in morphology is likely due to differences in the nucleation and growth rates of the LDHs, which are sensitive to the specific M^2+^ cation incorporated. Regarding crystal size, the Mg–Al, Ni–Al, and Zn–Al samples exhibited crystals of several micrometers. Conversely, the Co–Al sample consisted of finer crystals, approximately 500 nm in size, making them noticeably smaller than those of the other samples. Notably, XRD analysis indicated that the Zn–Al sample (Fig. 2d) formed a mixed-phase film containing both LDH and ZnO. Nevertheless, FE-SEM images still revealed the typical plate-like structure characteristic of LDH, suggesting that ZnO was either dispersed as fine particles within or on the surface of the LDH plates, or that the LDH crystals grew by incorporating a ZnO phase. The presence of this secondary phase is hypothesized to influence the overall compactness and long-term stability of the Zn–Al coating.

Observations of surface defects showed that the Mg–Al LDH coating (Fig. 2a) contained prominent cracks on the order of several micrometers. This suggests that the Mg–Al LDH film experienced greater difficulty in managing shrinkage and stress relaxation compared to the other coatings during fabrication or subsequent cooling. In stark contrast, no significant cracks were observed on the surfaces of the Co–Al, Ni–Al, or Zn–Al coatings. In particular, the Co–Al film, composed of fine crystals, likely benefited from enhanced stress dispersion, which effectively suppressed crack formation.

To quantify the film composition, EDS analysis was performed at multiple points for each coating, and the results are summarized in Table 2. The molar ratios of divalent cations to aluminum (M^2+^/Al) determined by EDS, the calculated *x* values (*x* = Al/[M^2+^ + Al]), and the *d*_003_ basal spacings of the LDH phases derived from XRD analysis are summarized in Table S1 in the SI. The molar fractions of trivalent cations (*x* = Al^3+^/[M^2+^ + Al^3+^]) for the (a) Mg–Al, (b) Co–Al, and (c) Ni–Al LDH systems were 0.16, 0.26, and 0.15, respectively. Typically, LDHs are represented by the general formula, which is generally stable within the range of 0.17 ≤ *x* ≤ 0.33.^26–28^ For the Mg, Co, and Ni systems, the *x* values are near this established range, confirming the formation of well-defined LDH structures. The *d*_003_ basal spacings (0.80 nm for Mg–Al, 0.87 nm for Co–Al, and 0.78 nm for Ni–Al) are intermediate between or close to the values reported for nitrate-type and carbonate-type LDHs, supporting the earlier interpretation that these films are predominantly nitrate-based with partial carbonate incorporation. In contrast, the (d) Zn–Al system exhibited a significantly lower *x* value of 0.05. This deviation is attributed to the formation of a mixed-phase coating [M_1−*x*_^2+^M_*x*_^3+^(OH)_2_]^*x*+^[A_*x*/*n*_^*n*−^]^*x*−^·*m*H_2_O, where the pure LDH phase comprising Zn–Al LDH and ZnO, as evidenced by the intense ZnO diffraction peaks in the XRD patterns (Fig. 1). In this system, the dehydration of Zn(OH)_2_—a primary building block for the brucite-like layers—leads to the competitive formation of ZnO. This process effectively reduces the amount of Zn species available for Al^3+^ substitution, thereby hindering the incorporation of trivalent cations into the LDH lattice. Consequently, the bulk composition appears Al-deficient, reflecting a physical mixture of a minor LDH phase and a dominant ZnO phase rather than a single-phase LDH with *x* = 0.05. Based on this stoichiometry, the phase fractions of LDH and ZnO in the Zn–Al coating were semi-quantitatively estimated. However, it should be noted that these estimates are approximate, with limitations arising from potential local compositional heterogeneity and the presence of any amorphous species not detected by XRD.

**Table 2:** Atomic compositions of the films prepared by steam coating at 140 °C for 12 h using an aqueous solution (pH = 10) containing either (a) Mg(NO_3_)_2_·6H_2_O, (b) Co(NO_3_)_2_·6H_2_O, (c) Ni(NO_3_)_2_·6H_2_O, or (d) Zn(NO_3_)_2_·6H_2_O (at%)

Samples	C	N	O	Al	Mg	Co	Ni	Zn
(a) Mg	2.1	2.9	64.6	5.0	26.7	—	—	—
(b) Co	5.7	1.6	71.4	5.5	—	15.6	—	—
(c) Ni	5.0	3.6	69.7	3.3	—	—	18.4	—
(d) Zn	4.5	5.5	55.8	1.0	—	—	—	19.7

**Fig. 3:**
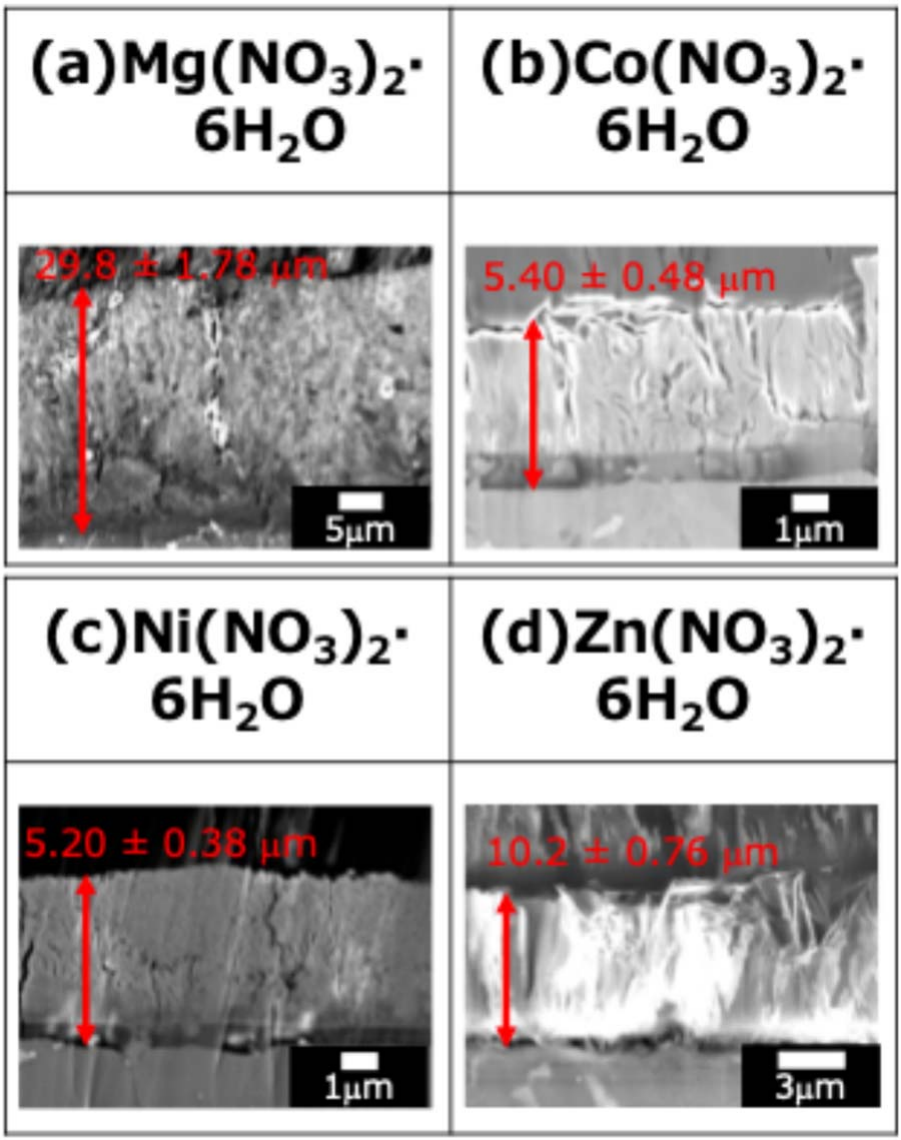
Cross-sectional SEM images of the films prepared by steam coating at 140 °C for 12 h using an aqueous solution (pH = 10) containing 1.0 M of (a) Mg(NO_3_)_2_·6H_2_O, (b) Co(NO_3_)_2_·6H_2_O, (c) Ni(NO_3_)_2_·6H_2_O, or (d) Zn(NO_3_)_2_·6H_2_O.

The cross-sectional SEM images of the fabricated LDH coatings are presented in Fig. 3. The film thickness was determined using a systematic statistical approach based on these images. For each specimen, five different observation areas were randomly selected. Within each area, the thickness was measured at 10 equidistant points. The reported film thickness represents the mean value of these 10 points. Notably, cracks and voids within the LDH layers were included in the average values to accurately reflect the effective thickness and the macroscopic growth behavior of the coatings, rather than focusing solely on the dense regions.

**Fig. 4:**
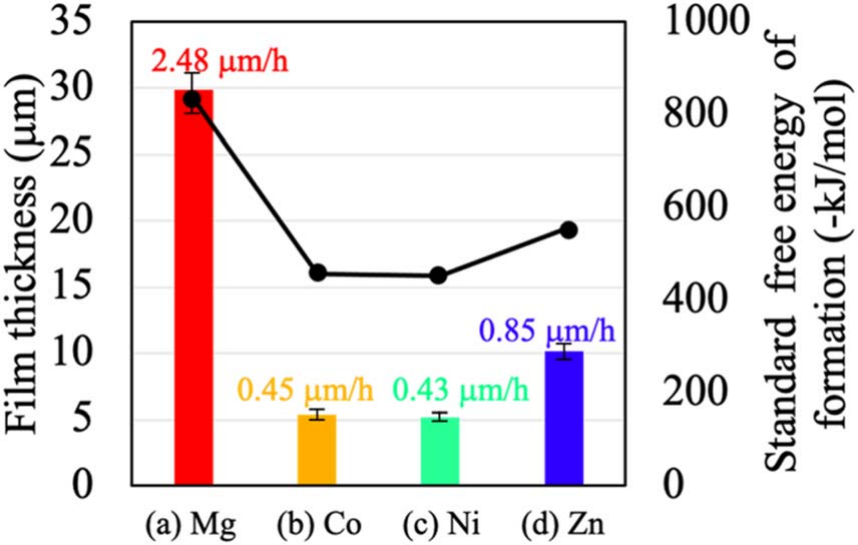
Thickness of films prepared by steam coating at 140 °C for 12 h using an aqueous solution (pH = 10) containing 1.0 M of (a) Mg(NO_3_)_2_·6H_2_O, (b) Co(NO_3_)_2_·6H_2_O, (c) Ni(NO_3_)_2_·6H_2_O, or (d) Zn(NO_3_)_2_·6H_2_O.

The relationship between the film thickness, deposition rate, and the standard Gibbs free energy of formation (Δ*G*^0^_f_) for the corresponding divalent metal hydroxides is summarized in Fig. 4. The standard Gibbs free energy of formation (Δ*G*^0^_f_) for the hydroxides of the respective divalent cations is known to be −834 kJ mol^−1^ for Mg(OH)_2_, −456 kJ mol^−1^ for Co(OH)_2_, −453 kJ mol^−1^ for Ni(OH)_2_, and −552 kJ mol^−1^ for Zn(OH)_2_.^29^ As shown in Fig. 4, the thickness of the fabricated films increased in the following order: Ni–Al (Ni(NO_3_)_2_) → Co–Al. (Co(NO_3_)_2_) → Zn–Al (Zn(NO_3_)_2_) → Mg–Al (Mg(NO_3_)_2_). When these film thickness data were compared with the standard Gibbs free energy of formation (Δ*G*^0^_f_), a clear tendency was confirmed: the film thickness increases as the absolute value of Δ*G*^0^_f_ increases (*i.e.*, the easier the hydroxide is to form and the more thermodynamically stable it is). Notably, Mg(OH)_2_ exhibited the lowest Δ*G*^0^_f_ value (−834 kJ mol^−1^) compared to the other hydroxides, and consequently, the Mg–Al LDH coating showed the largest film thickness. This strongly suggests a definitive correlation between the ease of hydroxide formation (thermodynamic stability) and the growth rate of the LDH film.

In the cross-sectional SEM images of all samples, the presence of fine cracks propagating from the substrate interface toward the film interior was observed. These cracks are presumed to be caused by internal stress resulting from the difference in the coefficients of thermal expansion between the Al–Si–Cu substrate and the LDH coating. In particular, the Mg–Al film exhibited more pronounced and larger cracks throughout the cross-section (Fig. 3a), consistent with the observations in the surface SEM images (Fig. 2a). This severe cracking is hypothesized to be due to the extremely low Δ*G*^0^_f_ of Mg(OH)_2_. This low value promotes an exceptionally fast rate of LDH nucleation and crystal growth, leading to the rapid accumulation of substantial internal stress within the film. This stress, compounded by the coefficient of thermal expansion (CTE) mismatch, resulted in the formation of through-film defects (cracks). Crucially, these large-scale defects are expected to provide facile access for aggressive electrolytes to the substrate, potentially compromising the coating's ability to offer long-term corrosion protection.

**Fig. 5:**
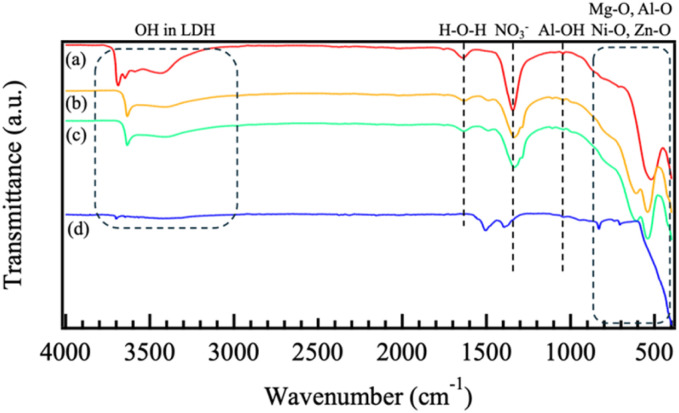
FT-IR spectra of films prepared by steam coating at 140 °C for 12 h using an aqueous solution (pH = 10) containing 1.0 M of (a) Mg(NO_3_)_2_·6H_2_O, (b) Co(NO_3_)_2_·6H_2_O, (c) Ni(NO_3_)_2_·6H_2_O, or (d) Zn(NO_3_)_2_·6H_2_O.

Fig. 5 shows the FT-IR spectra of (a) Mg–Al, (b) Co–Al, (c) Ni–Al, and (d) Zn–Al films. This analysis was performed to confirm the chemical bonding states, interlayer anions, and the presence of metal hydroxide species in the LDH films. In all spectra, absorption peaks around 420 cm^−1^ and 600 cm^−1^ were attributed to lattice vibrations of Al–O and the symmetric stretching of octahedral AlO_6_, respectively.^30^ An additional peak near 550 cm^−1^ was also assigned to Al–O lattice vibrations.^31^ Peaks observed approximately 450 cm^−1^, 570 cm^−1^, 460 cm^−1^, and 450 cm^−1^ were attributed to lattice vibrations of Mg–O, Co–O, Ni–O, and Zn–O, respectively, indicating the presence of metal hydroxide species corresponding to the divalent cations used as precursors.^25,26,32,33^ Regarding the interlayer anions, absorption bands were observed near 1350–1380 cm^−1^, and 1410 cm^−1^. While the peaks at 1380 cm^−1^ are characteristic of the *ν*_2_ stretching modes of intercalated NO_3_^−^ ions, the broad absorption in the 1350–1450 cm^−1^ region also overlaps with the asymmetric stretching of CO_3_^2−^ ions. Combined with the *d*_003_ spacing results from XRD, these spectra suggest that the formed films are predominantly nitrate-based LDHs but contain a significant amount of carbonate ions due to CO_2_ absorption from the atmosphere during the synthesis. A broad peak at approximately 3450 cm^−1^ was observed, corresponding to water molecules (H_2_O) in the LDH interlayers. Sharp peaks near 3650 cm^−1^ were attributed to highly crystalline hydroxides such as Mg(OH)_2_ and Co(OH)_2_.^27^ These peaks suggest the presence of M(OH)_2_ phases, either as part of the LDH brucite-like layers or, in the case of Zn–Al, as residual Zn(OH)_2_ due to incomplete dehydration to ZnO as indicated by XRD. Overall, these results indicate that, although NO_3_^−^ from the precursor solution was successfully intercalated, the resulting coatings are mixed-anion LDHs containing both NO_3_^−^ and CO_3_^2−^.

**Fig. 6:**
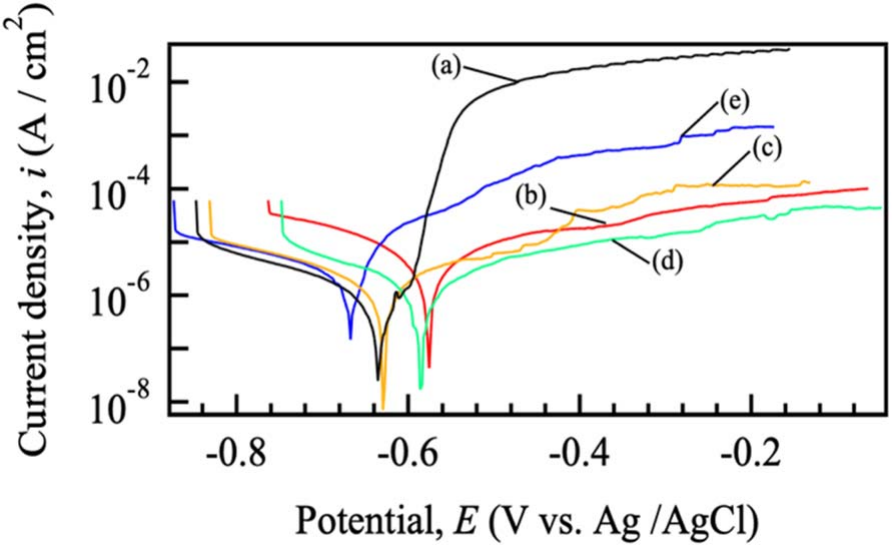
Polarization curves of (a) bare ADC12, and LDH-coated samples prepared with different divalent cations: (b) Mg–Al, (c) Co–Al, (d) Ni–Al, and (e) Zn–Al.

**Fig. 7:**
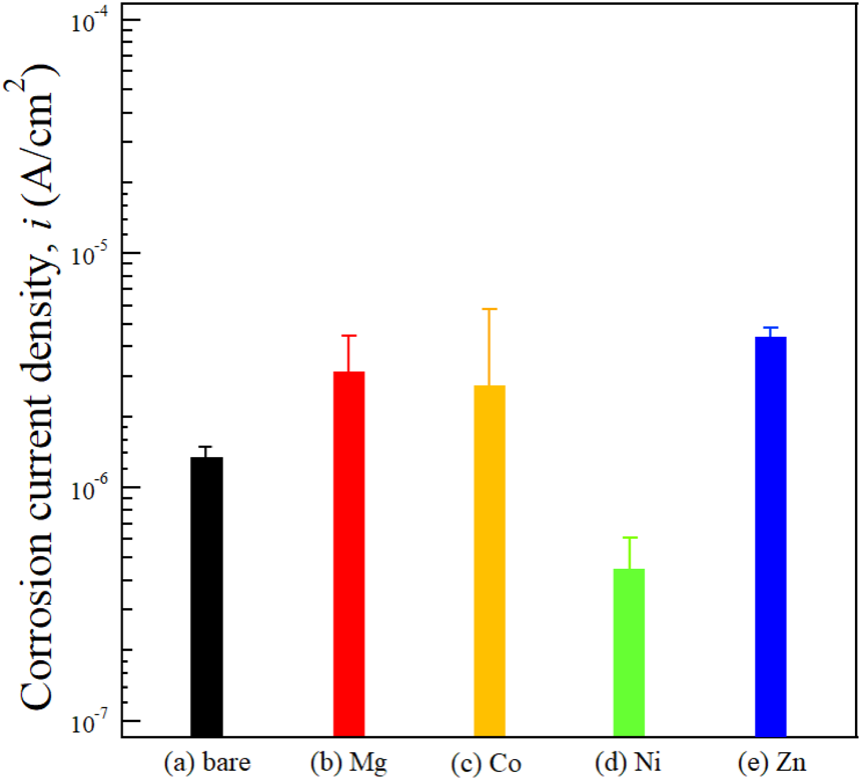
Corrosion current density values of (a) bare ADC12, and LDH-coated samples prepared with different divalent cations: (b) Mg–Al, (c) Co–Al, (d) Ni–Al, and (e) Zn–Al.

Fig. 6 shows the representative polarization curves of (a) the untreated ADC12 alloy and (b) Mg–Al, (c) Co–Al, (d) Ni–Al, and (e) Zn–Al LDH films. The electrochemical parameters, including the mean corrosion potential (*E*_corr_) and corrosion current density (*i*_corr_) based on triplicate measurements, are summarized in Fig. S2 and Table S2. The *E*_corr_ of the untreated alloy was −0.64 V, while the mean *E*_corr_ values for the Mg–Al, Co–Al, Ni–Al, and Zn–Al LDH films were −0.621 ± 0.042 V, −0.616 ± 0.045 V, −0.659 ± 0.055 V, and −0.671 ± 0.013 V, respectively. As shown in the polarization curves in Fig. 6, although the Mg–Al and Co–Al LDH coatings exhibited a slight positive shift in the mean *E*_corr_, their relatively large standard deviations—which reflect the intrinsic variability of the coatings caused by surface cracks and heterogeneity (as seen in Fig. 2 and 3)—suggest that the thermodynamic corrosion tendency is sensitive to local film defects. In contrast, the Ni–Al and Zn–Al LDH films showed slightly more negative *E*_corr_ values than the untreated alloy. Despite these varied shifts in *E*_corr_, all LDH-coated samples exhibited a distinct decrease in the anodic current densities, indicating the formation of a protective layer. Notably, only the Ni–Al system showed a simultaneous reduction in *i*_corr_ compared to the bare ADC12 (Fig. 7). While the mean *i*_corr_ for the Mg–Al, Co–Al, and Zn–Al coatings was higher than that of the untreated alloy due to the aforementioned structural defects, the emergence of pseudo-passive behavior in the anodic region of all LDH-coated samples demonstrates that these films effectively provide a physical barrier that kinetically suppresses the corrosion process, rather than relying on a noble-shift of the equilibrium potential.

The corrosion current density (*i*_corr_) of the untreated alloy was 1.18 × 10^−6^ A cm^−2^. As summarized in Fig. 7, S2, and Table S2, the averaged *i*_corr_ values for the Mg–Al, Co–Al, Ni–Al, and Zn–Al LDH coatings were found to be (2.65 ± 1.77) × 10^−6^ A cm^−2^, (2.39 ± 3.39) × 10^−6^, (3.89 ± 2.20) × 10^−7^, and (3.93 ± 0.91) × 10^−6^ A cm^−2^, respectively. Comparison of these mean values indicates that only the Ni–Al coating significantly reduced the corrosion current density to approximately 33% of that of the untreated alloy, thereby exhibiting superior corrosion resistance compared to all other specimens. This improvement is consistent with the study by Tedim *et al.*, which demonstrated that well-developed LDH conversion films can serve as an effective physical barrier to significantly reduce the *i*_corr_ of aluminum alloys.^34^ In contrast, the mean *i*_corr_ values of the Co–Al, Mg–Al, and Zn–Al LDH coatings were higher than that of the untreated alloy. Notably, the Co–Al coating showed a very large standard deviation, with its mean *i*_corr_ exceeding that of the bare alloy. This indicates that the Co–Al LDH film does not provide consistent or reliable corrosion protection across replicates, despite its seemingly dense appearance in surface observations. The *i*_corr_ values of the LDH-coated samples generally increased in the order of Ni–Al < Co–Al < Mg–Al < Zn–Al. In particular, all LDH coatings except for Ni–Al exhibited higher mean *i*_corr_ values and larger standard deviations than the untreated alloy. This significant variability and higher current density can be attributed to the intrinsic heterogeneity of these coatings, particularly the presence of surface and cross-sectional cracks (as observed in Fig. 2 and 3), which facilitated localized electrolyte penetration to the substrate. Even for the Co–Al coating, which appeared dense in SEM, the large standard deviation suggests the presence of stochastic defects that compromise its barrier consistency. According to the growth mechanism of LDH on aluminum surfaces discussed by Lin *et al.*, the direct growth process involves complex dissolution–precipitation steps.^35^ The formation of these micro-cracks in our samples might be attributed to the internal stresses generated by the differing growth rates of the LDH layers containing various divalent cations.

Despite the higher mean *i*_corr_ for several specimens compared to the bare alloy, it is noteworthy that all LDH coatings displayed stable passive-like behavior in the anodic region. This indicates that while the initial corrosion current at *E*_corr_ was relatively high due to local defects (cracks), the LDH layers partially functioned as physical barriers that suppressed rapid anodic dissolution compared to the bare alloy at higher potentials. In the anodic scan from *E*_corr_, the untreated alloy showed a rapid increase in current density at approximately −0.60 V, with the rate of increase slowing only near −0.52 V. In contrast, all LDH coatings exhibited more consistent passive-like behavior, demonstrating their ability to act as physical barriers that kinetically hinder the progression of the anodic reaction once a passive film or barrier layer is stabilized. In the cathodic region, all curves showed a sharp rise in current density attributed to the hydrogen evolution reaction (HER), although the onset potentials differed, likely due to the influence of various divalent cations in the coatings on the hydrogen overpotential.

**Fig. 8:**
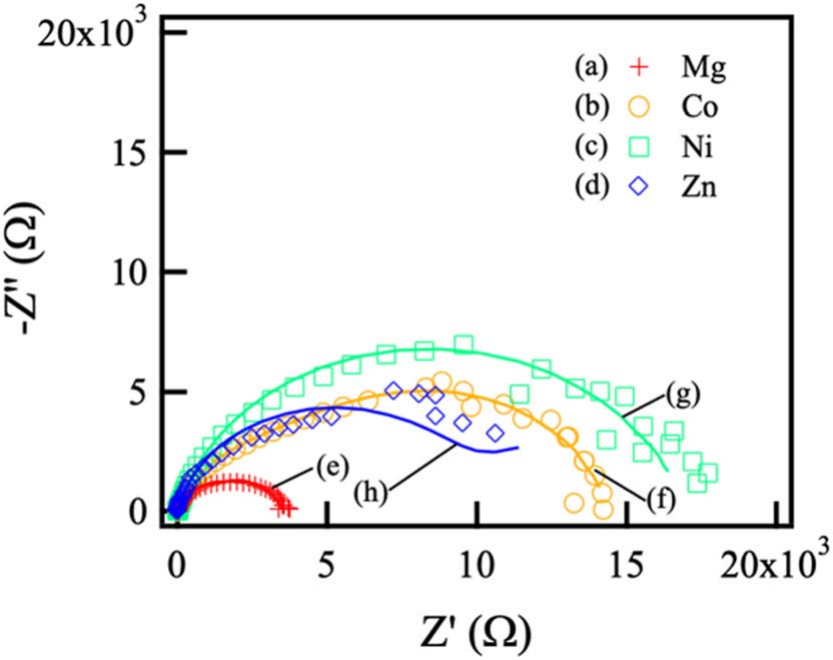
Nyquist diagrams and fitting results of LDH-coated samples prepared with different divalent cations: (a) Mg–Al, (b) Co–Al, (c) Ni–Al, and (d) Zn–Al.

Fig. 8 shows the Nyquist plots of (a) Mg–Al, (b) Co–Al, (c) Ni–Al, and (d) Zn–Al films, along with the corresponding fitting results for (e) Mg–Al, (f) Co–Al, (g) Ni–Al, and (h) Zn–Al films. The measured data are represented by symbols, and the solid lines indicate fitted curves.

**Fig. 9:**
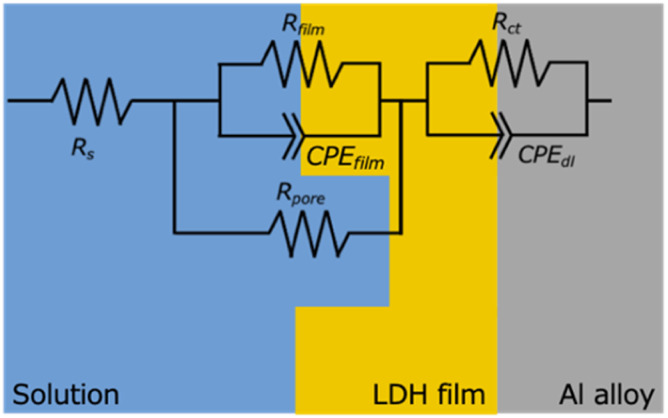
Equivalent circuit model used for fitting the EIS data. *R*_s_ represents the solution resistance; *R*_film_ and CPE_film_ are the resistance and constant phase element of the LDH film, respectively; *R*_pore_ is the resistance of the electrolyte within the film pores; *R*_ct_ and CPE_dl_ are the charge transfer resistance and double-layer capacitance at the substrate/electrolyte interface. This model was applied to all LDH-coated samples (Mg–Al, Co–Al, Ni–Al, and Zn–Al).

To qualitatively analyze the obtained EIS responses, fitting was performed using the equivalent circuit and the corresponding physical model as shown in Fig. 9. In this circuit, *R*_s_ represents the solution resistance; *R*_film_ and CPE_film_ correspond to the resistance and capacitive element of the film, respectively; *R*_pore_ represents the pore resistance; and *R*_ct_ and CPE_dl_ represent the charge transfer resistance and double-layer capacitance at the substrate/film interface. The values of each element obtained from the fitting are listed in Table 3. It should be noted that, due to the lack of replicate EIS measurements caused by instrument failure, these parameters are intended to provide qualitative insight into the barrier properties and should not be interpreted as statistically definitive values.

**Table 3:** Electrochemical parameters obtained from EIS fitting for various LDH-coated samples: (a) Mg–Al, (b) Co–Al, (c) Ni–Al, and (d) Zn–Al films. These values are derived from representative spectra; due to instrument failure, these parameters are presented as qualitative indicators of the physical barrier properties rather than statistically averaged values. The fitting errors were below 10%

Samples	*R*_s_ (Ω cm^2^)	*R*_film_ (Ω cm^2^)	CPE_film_ (Ω^−1^ cm^−2^ s^*n*^)	*R*_pore_ (Ω cm^2^)	*R*_ct_ (Ω cm^2^)	CPE_dl_ (Ω^−1^ cm^−2^ s^*n*^)
(a) Mg	29.7	3.95 × 10^3^	4.31 × 10^−5^	2.10 × 10^3^	1.86 × 10^3^	3.43 × 10^−5^
(b) Co	13.5	1.47 × 10^4^	3.86 × 10^−5^	1.39 × 10^4^	1.02 × 10^3^	9.13 × 10^−5^
(c) Ni	11.0	2.12 × 10^4^	3.14 × 10^−5^	2.59 × 10^4^	3.04 × 10^3^	5.88 × 10^−4^
(d) Zn	9.8	1.04 × 10^4^	1.24 × 10^−5^	1.56 × 10^4^	1.92 × 10^3^	3.05 × 10^−4^

The *R*_film_ values obtained from the representative spectra suggest a trend where the LDH coatings generally provide a physical barrier compared to the untreated alloy. However, the *R*_film_ values do not strictly follow the *i*_corr_ order obtained from the statistically validated polarization curves (Fig. S2 and Table S2). This discrepancy is likely because *R*_film_ represents the physical integrity of the coating layer itself, whereas *i*_corr_ reflects the overall electrochemical kinetics at the interface. For instance, the Zn–Al LDH coating exhibited a relatively high *R*_film_ value despite its high *i*_corr_. This can be attributed to the mixed-phase Zn–Al LDH/ZnO coating identified by XRD (Fig. 1); while the ZnO phase contributes to the physical barrier effect, its semiconductor-like behavior may facilitate charge transfer compared to a purely insulating film, thereby leading to a higher *i*_corr_ despite the increased *R*_film_.

Although thicker films are generally expected to provide higher barrier properties, the Mg–Al LDH coating, which formed the thickest film, did not exhibit a proportionally high *R*_film_. This is likely due to the rapid crystal growth caused by the low standard Gibbs free energy of formation (Δ*G*^0^_f_) of Mg(OH)_2_, which led to stress accumulation and the formation of large cracks within the film. These structural defects likely acted as pathways for electrolyte penetration, preventing *R*_film_ from reaching values seen in denser films and resulting in a higher *i*_corr_ as supported by the triplicate polarization data. In contrast, the Ni–Al and Co–Al LDH coatings formed dense films (as observed in SEM images), which qualitatively align with their improved barrier properties. The Ni–Al coating performed better than the Co–Al coating, which can be attributed to the wider bandgap of Ni(OH)_2_ (3.6 eV) compared to Co(OH)_2_ (2.8 eV), leading to reduced charge transfer and higher resistance.^36,37^

In conclusion, while the EIS fitting parameters (Table 3) provide useful physical models for the LDH-coated systems, the definitive corrosion resistance ranking is based on the statistical mean values of *i*_corr_ and ion-release behavior, which consistently follow the order: Ni–Al > Co–Al > Mg–Al > Zn–Al.

**Fig. 10:**
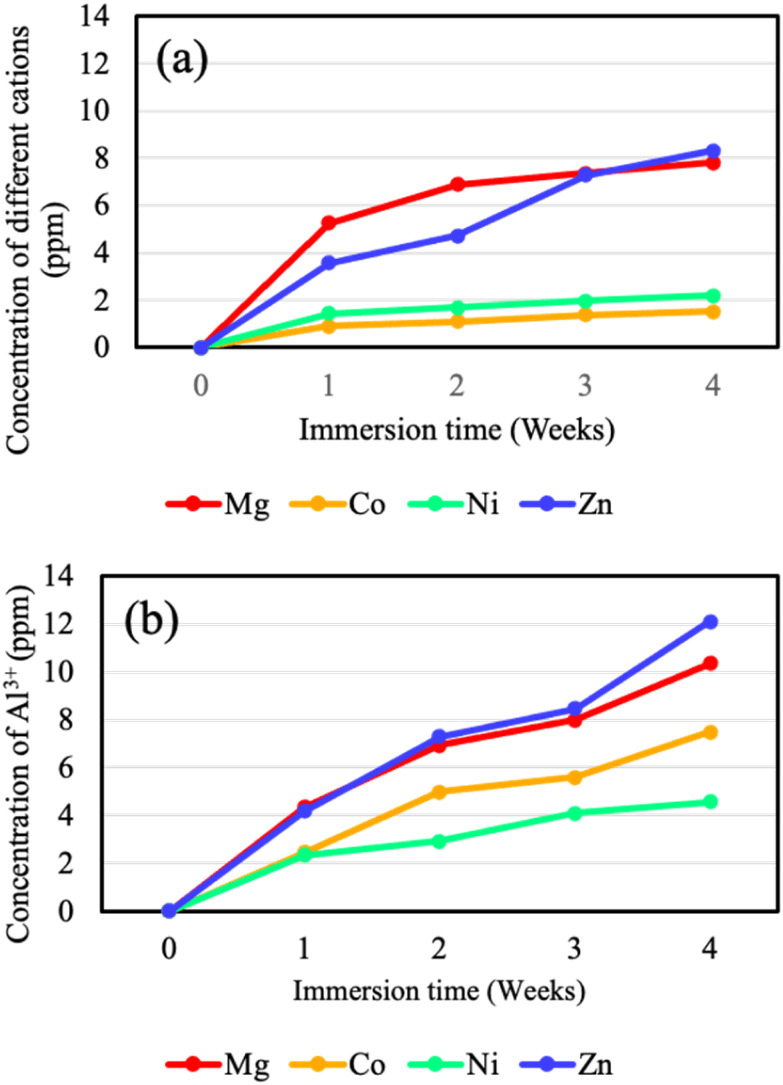
Change in the concentration of (a) divalent cations (M^2+^) and (b) Al^3+^ ions dissolved into the 5.0 wt% NaCl solution as a function of immersion time. All values were obtained from a defined surface area of 1.0 cm^2^.

Fig. 10 shows the relationship between immersion time and the concentrations of divalent cations (M^2+^), and Al^3+^ released into 5.0 wt% NaCl solution from each LDH coating. The average values and standard deviations calculated from triplicate measurements are summarized in Table S3. After 4 weeks of immersion, the release of divalent cations followed the order: Zn–Al > Mg–Al > Ni–Al > Co–Al LDH. The relatively high M^2+^ release from Mg–Al and Zn–Al LDH indicates that these coatings are thermodynamically less stable in NaCl solution and are more susceptible to structural degradation. In contrast, Ni–Al and Co–Al LDH exhibited significantly lower M^2+^ release, suggesting higher chemical stability in NaCl solution during long-term immersion.

The Al^3+^ release after 4 weeks of immersion followed the order: Zn–Al > Mg–Al > Co–Al > Ni–Al LDH. In LDH, divalent cations are generally more abundant than trivalent cations, with a typical M^3+^/M^2+^ ratio of 1/6 < M^3+^/M^2+^ < 1/3.^17–19,38,39^ However, in this study, the released amount of Al^3+^ was higher than that of M^2+^. This indicates that, in addition to cation release from the LDH coating itself, a substantial portion of Al^3+^ originated from the corrosion of the underlying Al substrate through local defects in the coating. Therefore, a higher Al^3+^ release corresponds to lower barrier performance and reduced corrosion resistance.

Based on these long-term immersion results, the overall corrosion resistance (defined by ion-release stability) is ranked as follows: Ni–Al > Co–Al > Mg–Al > Zn–Al LDH. It should be noted, however, that while the Co–Al and Ni–Al LDH coatings showed minimal ion release, the electrochemical results revealed that the Co–Al coating exhibited high variability and a mean *i*_corr_ exceeding that of the untreated alloy. This suggests that while the Co–Al coating is chemically stable over long periods, its initial barrier performance can be inconsistent due to stochastic local defects.

**Fig. 11:**
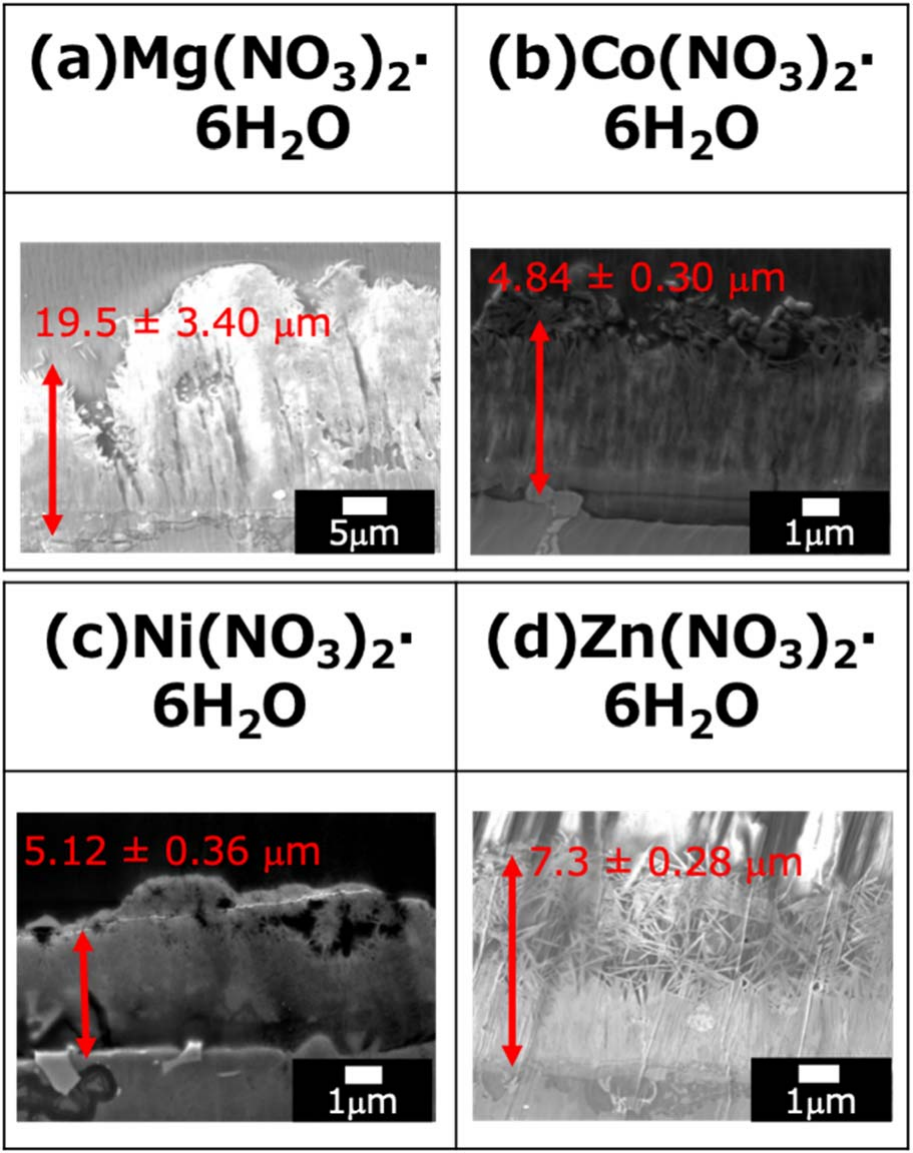
Cross-sectional SEM images of (a) Mg–Al, (b) Co–Al, (c) Ni–Al, and (d) Zn–Al films after 4 weeks of immersion in 5.0 wt% NaCl solution.

Fig. 11 shows cross-sectional SEM images of (a) Mg–Al, (b) Co–Al, (c) Ni–Al, and (d) Zn–Al films after 4 weeks of immersion in 5.0 wt% NaCl solution. The film thickness decreased after immersion for all samples; specifically, the reduction rates for the Mg–Al, Co–Al, Ni–Al, and Zn–Al LDH coatings were estimated to be 34.6%, 7.7%, 5.6%, and 28.5%, respectively. The sequence of thickness reduction (Ni–Al < Co–Al < Zn–Al < Mg–Al) reflects the overall long-term stability and aligns with the trends inferred from M^2+^ and Al^3+^ release. The high reliability of these conclusions is supported by the quantitative ICP-OES data and cross-sectional SEM observations, both of which were performed in triplicate (*n* = 3) to ensure statistical robustness.

The concentrations of dissolved metal ions and the reduction rates of the LDH coatings followed the order of Ni–Al < Co–Al < Zn–Al < Mg–Al. This trend is partially consistent with the trends observed in the mean *i*_corr_ values (Fig. S3 and Table S4), specifically in identifying Ni–Al as the most protective and Mg–Al/Zn–Al as the least stable. However, as noted previously, the Co–Al system exhibited a large standard deviation in *i*_corr_ across replicates, contrasting with its relatively uniform appearance and low dissolution rate. While the representative EIS spectra (Table 3) provide qualitative support by showing higher *R*_film_ and *R*_ct_ for the Ni–Al and Co–Al systems, the combined ICP/SEM data—based on independent replicates—serve as the primary evidence for the superior long-term barrier performance of the Ni–Al coatings.

In the coatings with higher thickness reduction, namely (a) Mg–Al and (d) Zn–Al LDH, degradation was observed not only on the surface but also internally, including near the film/substrate interface. This suggests that the pre-existing cracks in the Mg–Al coating (Fig. 2 and 3) and the presence of the ZnO mixed phase in the Zn–Al coating provided pathways for electrolyte penetration, accelerating film dissolution and substrate corrosion. In contrast, the (c) Ni–Al LDH coatings exhibited the most uniform degradation, with no significant local defects. The Co–Al LDH also showed low thickness reduction; however, its protective reliability is limited by the variability observed in electrochemical tests.

The superior performance of Ni–Al LDH, despite its lower initial thickness compared to Mg–Al LDH, can be attributed to the higher structural density and suppressed crack formation. While Mg–Al LDH grows rapidly to form a thick layer, it develops a more porous structure that allows electrolyte infiltration. In contrast, Ni^2+^ cations facilitate the formation of a more compact LDH lattice, which acts as a more effective physical barrier against Cl^−^ ions.

## 4. Conclusions

In this study, nitrate-based layered double hydroxide (LDH) coatings with different divalent cations (M^2+^)—Mg–Al, Co–Al, Ni–Al, and Zn–Al—were fabricated on Al–Si–Cu alloy substrates using a low-environmental-impact steam coating method. Structural and spectroscopic analyses confirmed that the coatings possessed an LDH structure containing both NO_3_^−^ and CO_3_^2−^ as interlayer anions, though NO_3_^−^ was the primary species from the precursor. Additionally, XRD analysis revealed that the Zn–Al coating consisted of a mixed phase of LDH and ZnO.

The corrosion resistance of the LDH-coated Al–Si–Cu alloy was assessed *via* polarization tests, electrochemical impedance spectroscopy (EIS), and immersion tests in saline solution (monitoring metal ion release). Based on the mean corrosion current density (*i*_corr_) and long-term ion-release behavior (*n* = 3), the overall corrosion resistance was ranked in the order: Ni–Al > Co–Al > Mg–Al > Zn–Al LDH. This sequence is consistent with the chemical stability observed during long-term immersion, although the initial electrochemical barrier performance of the Co–Al, Mg–Al, and Zn–Al coatings was compromised by stochastic defects, resulting in mean *i*_corr_ values higher than those of the untreated alloy.

The Zn–Al and Mg–Al coatings exhibited the highest mean *i*_corr_ values and significant experimental variability. In the case of Mg–Al, this was due to large cracks from rapid crystal growth, while for Zn–Al, the presence of the ZnO mixed phase contributed to higher dissolution. These structural defects acted as pathways for electrolyte penetration, significantly reducing the barrier performance.

In contrast, the Ni–Al coating consistently demonstrated the most superior and reproducible performance, as evidenced by the lowest mean *i*_corr_ and high *R*_film_ values across all replicates. Although the Co–Al coating appeared dense in SEM and showed low ion release over long periods, its initial *i*_corr_ was reported at approximately 10^−6^ A cm^−2^ with a very large standard deviation, exceeding the mean value of the bare alloy. This highlights that while Co–Al LDH is chemically stable, its protection can be inconsistent compared to the Ni–Al system.

This study demonstrated that the primary factor determining the corrosion resistance of LDH coatings was not the film thickness, but rather the coating density and the absence of structural heterogeneity. The findings emphasized that strict alignment with replicate statistics—considering both mean values and standard deviations—was essential for accurately evaluating the protective performance of LDH-based coatings.

## Author contributions

Conceptualization and methodology, T. I.; experimental and data analysis, I. M., Y. A., H. O., and K. F.; writing—original draft preparation, I. M. and T. I.; writing—review and editing, T. I.; supervision, T. I.; project administration and funding acquisition, T. I. All authors have read and agreed to the published version of the manuscript.

## Conflicts of interest

There are no conflicts to declare.

## Supplementary Material

RA-016-D5RA09199C-s001

## Data Availability

The data supporting this article have been included as part of the supplementary information (SI). The primary electrochemical datasets are available upon request from the corresponding author. Supplementary information: the details of Experimental procedure, FE-SEM images, summary of M^2+^/Al ratios (EDS), calculated x values (x = Al/[M^2+^ + Al]), and XRD-derived *d*_003_ spacings for the Mg–Al, Co–Al, Ni–Al, and Zn–Al LDH systems, polarization curves, summary of *E*_corr_ and *i*_corr_ values calculated from Tafel plots of polarization curves (SI 4) for (a) Mg–Al, (b) Co–Al, (c) Ni–Al, and (d) Zn–Al LDH films, and time-dependent concentrations of metal ions dissolved into a 5.0 wt.% NaCl solution from (a–d) M^2+^ (M = Mg, Co, Ni, Zn) and (e–h) Al^3+^ components in Mg–Al, Co–Al, Ni–Al, and Zn–Al alloys. See DOI: https://doi.org/10.1039/d5ra09199c.
