# Supplementary material for: Preparation and characterization of layered double hydroxide (LDH) films with varying divalent cation species on Al–Si–Cu alloys by steam coating
Source: RSC Adv. 2026 Apr 13;16(21):19176–86. doi: 10.1039/d5ra09199c (PMC13071577; doi:10.1039/d5ra09199c)
Supplement: RA-016-D5RA09199C-s001 [file RA-016-D5RA09199C-s001.pdf]

Supplementary Information (SI) for RSC Advances.
This journal is © The Royal Society of Chemistry 2026

Electronic Supplementary Information

Preparation and Characterization of Layered Double Hydroxide (LDH) Films with Varying Divalent Cation Species on Al-Si-Cu Alloy by Steam Coating

Io Matsui,[a] Yuki Atsuumi,[a] Hikari Ouchi,[a] Kota Fukuhara,[a] Takahiro Ishizaki*[b]

[a]Materials Science and Engineering, Graduate School of Engineering and Science, Shibaura Institute of Technology, Tokyo 135-8548, Japan.
[b]College of Engineering, Shibaura Institute of Technology, Tokyo 135-8548, Japan. E-mail: ishizaki@shibaura-it.ac.jp

S1. Experimental Section

To evaluate the concentration of dissolved metal ions after the hydrothermal process, the following analytical protocol was employed:

- **Sampling and Pre-treatment:** After the designated reaction time (12–48 h), the autoclave was cooled, and the entire liquid phase (approx. 15 mL) was collected. To prevent further precipitation or adsorption of ions onto the vessel walls, the collected solution was immediately acidified to pH < 2 using ultra-pure HNO_3.
- **Filtration and Evaporation Control:** The acidified solution was filtered through a 0.1 μm membrane filter to remove any suspended substance. The volume loss due to evaporation was found to be negligible (< 2%); however, the final volume was precisely adjusted using ultrapure water to ensure accurate concentration calculations.
- **ICP-OES Analysis:** Metal ion concentrations (Mg, Co, Ni, Zn, and Al) were determined using an ICP-OES (Agilent 5110). A five-point calibration curve was established for each element, ranging from 0.1 to 10 mg/L, with a correlation coefficient (R^2) > 0.999.
- **Detection Limits and Normalization:** The limits of detection (LOD) and quantification (LOQ) were determined based on the standard deviation of ten blank measurements (3σ and 10 σ, respectively).
- **Uncertainty:** All measurements were performed in triplicate, and the data are reported as the mean value ± standard deviation in ESI 5 to represent experimental uncertainty."

Figure S1: FE-SEM images of the LDH films, taken from two different locations on the same substrate, prepared by steam coating at 140 ^{o}C for 12 h using an aqueous solution (pH = 10) containing 1.0 M of (I) First Position and (II) Second Position. (a) $Mg(NO_3)_2 \cdot 6H_2O$, (b) $Co(NO_3)_2 \cdot 6H_2O$, (c) $Ni(NO_3)_2 \cdot 6H_2O$, or (d) $Zn(NO_3)_2 \cdot 6H_2O$. These supplementary images confirm the morphological homogeneity across the coated surface, supporting the representativeness of the data shown in the main text (Fig. 2).

(I)

	(a)$Mg(NO_3)_2 \cdot 6H_2O$	(b)$Co(NO_3)_2 \cdot 6H_2O$	(c)$Ni(NO_3)_2 \cdot 6H_2O$	(d)$Zn(NO_3)_2 \cdot 6H_2O$
**×500**	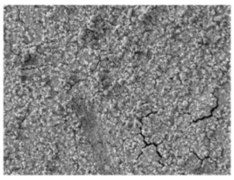	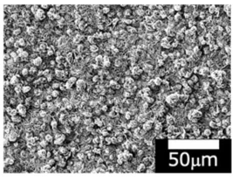	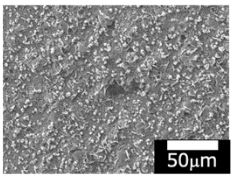	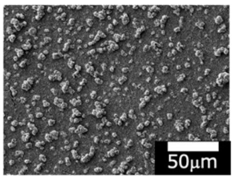
**×10000**	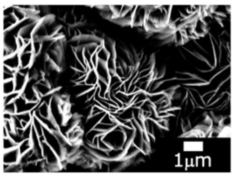	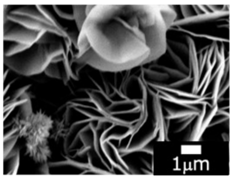	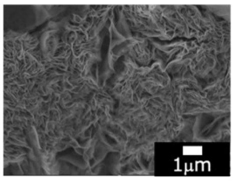	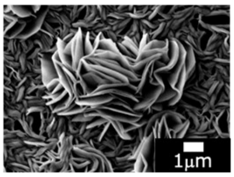

(II)

	(a)$Mg(NO_3)_2 \cdot 6H_2O$	(b)$Co(NO_3)_2 \cdot 6H_2O$	(c)$Ni(NO_3)_2 \cdot 6H_2O$	(d)$Zn(NO_3)_2 \cdot 6H_2O$
**×500**	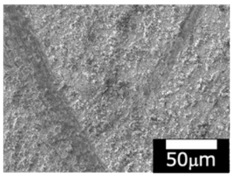	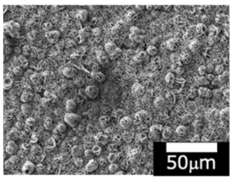	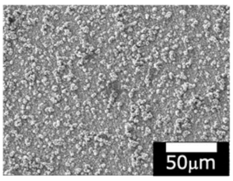	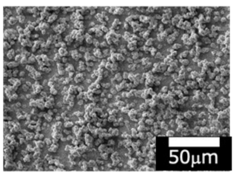
**×10000**	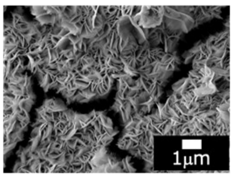	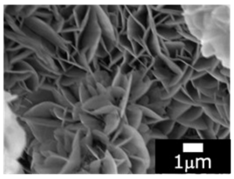	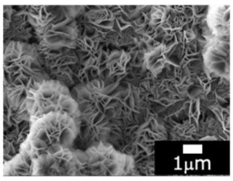	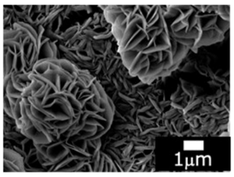

Figure S2. Polarization curves of LDH-coated samples prepared with different divalent cations: (a) Mg–Al, (b) Co–Al, (b) Ni–Al, and (d) Zn–Al. All black solid lines in all figures show the polarization curves of bare ADC 12. All polarization measurements were conducted in 5 wt.% NaCl aqueous solution at room temperature three times.

(a) Mg-Al LDH

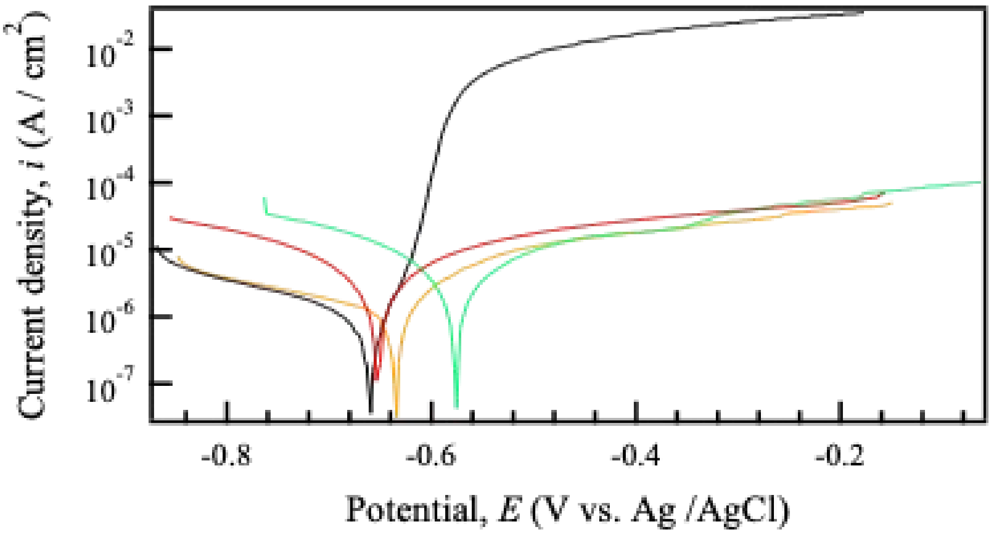

(b) Co-Al LDH

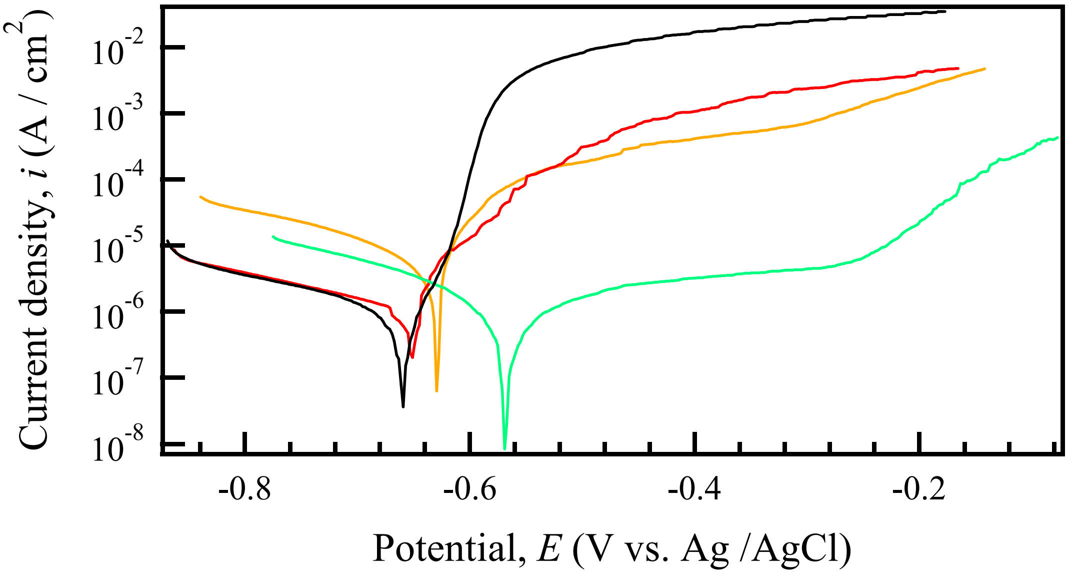

(c) Ni-Al LDH

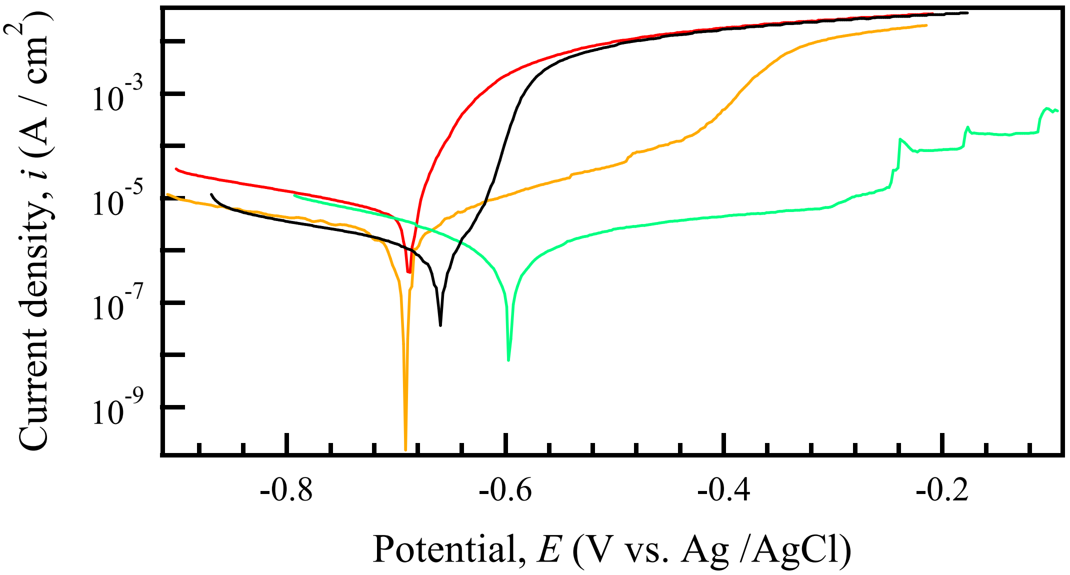

(d) Zn-Al LDH

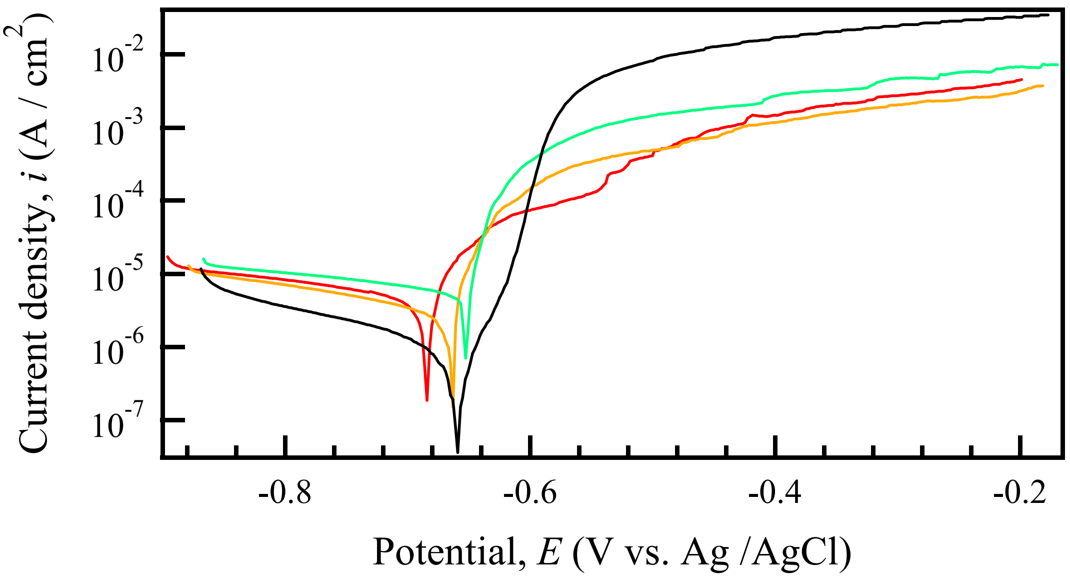

Table S1: Summary of M^{2+}/Al ratios (EDS), calculated x values (x = Al/[M^{2+}+Al]), and XRD-derived d_{003} spacings for the Mg–Al, Co–Al, Ni–Al, and Zn–Al LDH systems.

	M^{2+}/Al ratios	Calculated x values	XRD-derived d_{003} spacing values (nm)
(a) Mg-Al	5.34±0.45	0.16±0.02	0.80
(b) Co–Al	2.84±0.22	0.26±0.03	0.87
(c) Ni–Al	5.58±0.48	0.15±0.02	0.78
(d) Zn-Al	19.7±1.8	0.05±0.01	0.76

Table S2 Summary of E_{corr} and i_{corr} values calculated from Tafel plots of polarization curves (Fig. S3) for (a) Mg-Al, (b) Co-Al, (c) Ni-Al, and (d) Zn-Al LDH films. All quantitative data in Table S4 represent the mean values obtained from three independently prepared specimens (n = 3) to ensure reproducibility.

(a) Mg-Al	E_{corr} (V)	i_{corr} (A/cm^2)
1st	-0.653	4.66×10^{-6}
2nd	-0.635	1.98×10^{-6}
3rd	-0.574	1.32×10^{-6}
Ave.	-0.621±0.042	$(2.65\pm1.77)\times10^{-6}$

(b) Co-Al	E_{corr} (V)	i_{corr} (A/cm^2)
1st	-0.656	5.29×10^{-7}
2nd	-0.626	6.30×10^{-6}
3rd	-0.567	3.34×10^{-7}
Ave.	-0.616±0.045	$(2.39\pm3.39)\times10^{-6}$

(c) Ni-Al	E_{corr} (V)	i_{corr} (A/cm^2)
1st	-0.692	6.23×10^{-7}
2nd	-0.69	3.58×10^{-7}
3rd	-0.595	1.87×10^{-7}
Ave.	-0.659±0.055	$(3.89\pm2.20)\times10^{-7}$

(d) Zn-Al	E_{corr} (V)	i_{corr} (A/cm^2)
1st	-0.685	3.19×10^{-6}
2nd	-0.669	3.66×10^{-6}
3rd	-0.66	4.95×10^{-6}
Ave.	-0.671±0.013	$(3.93\pm0.91)\times10^{-6}$

Table S3 Time-dependent concentrations of metal ions dissolved into a 5.0 wt.% NaCl solution from (a–d) M^{2+} (M = Mg, Co, Ni, Zn) and (e–h) Al^{3+} components in Mg-Al, Co-Al, Ni-Al, and Zn-Al alloys.

All quantitative data in Table ESI 5 represent the mean values obtained from three independently prepared specimens (n = 3) to ensure reproducibility.

(a) Mg^{2+} in Mg-Al	0	1 week	2 weeks	3 weeks	4 weeks
1st	0	5.25	6.88	7.35	7.80
2nd	0	5.33	6.95	7.52	7.99
3rd	0	5.18	6.78	7.21	7.68
Ave.	0	5.25±0.08	6.87±0.09	7.36±0.16	7.82±0.16

(b) Co^{2+} in Co-Al	0	1 week	2 weeks	3 weeks	4 weeks
1st	0	0.89	1.09	1.37	1.53
2nd	0	1.01	1.25	1.52	1.65
3rd	0	0.77	0.99	1.23	1.44
Ave.	0	0.89±0.12	1.11±0.13	1.37±0.15	1.54±0.11

(c) Ni^{2+} in Ni-Al	0	1 week	2 weeks	3 weeks	4 weeks
1st	0	1.43	1.69	1.96	2.20
2nd	0	1.56	1.85	2.11	2.35
3rd	0	1.29	1.49	1.83	2.01
Ave.	0	1.43±0.14	1.68±0.18	1.97±0.14	2.19±0.17

(d) Zn^{2+} in Zn-Al	0	1 week	2 weeks	3 weeks	4 weeks
1st	0	3.56	4.72	7.28	8.32
2nd	0	3.68	4.95	7.45	8.55
3rd	0	3.41	4.66	7.08	8.11
Ave.	0	3.55±0.14	4.78±0.15	7.27±0.19	8.33±0.22

(e) Al^{3+} in Mg-Al	0	1 week	2 weeks	3 weeks	4 weeks
1st	0	4.35	6.94	7.99	10.35
2nd	0	4.52	7.15	8.18	10.58
3rd	0	4.13	6.82	7.78	10.18
Ave.	0	4.33±0.20	6.97±0.17	7.98±0.20	10.37±0.20

(f) Al^{3+} in Co-Al	0	1 week	2 weeks	3 weeks	4 weeks
1st	0	2.45	4.99	5.58	7.49
2nd	0	2.61	5.19	5.75	7.68
3rd	0	2.31	4.81	5.45	7.31
Ave.	0	2.46±0.15	5.00±0.19	5.59±0.15	7.49±0.19

(g) Al^{3+} in Ni-Al	0	1 week	2 weeks	3 weeks	4 weeks
1st	0	2.33	2.92	4.09	4.56
2nd	0	2.45	3.09	4.25	4.78
3rd	0	2.28	2.78	3.89	4.48
Ave.	0	2.35±0.09	2.93±0.16	4.08±0.18	4.61±0.16

(h) Al^{3+} in Zn-Al	0	1 week	2 weeks	3 weeks	4 weeks
1st	0	4.16	7.28	8.47	12.12
2nd	0	4.32	7.39	8.68	12.43
3rd	0	4.01	7.15	8.30	12.00
Ave.	0	4.16±0.16	7.27±0.12	8.48±0.19	12.18±0.22
